# Clinicopathologic and Prognostic Significance of Thymopoietin-α Overexpression in Gastric Cancer

**DOI:** 10.7150/jca.30738

**Published:** 2019-08-28

**Authors:** Ding-Ping Sun, Phui-Ly Liew, Chih-Chan Lin, Shih-Ting Hung, Tai-Chi Chen, Chia-Lang Fang, Kai-Yuan Lin

**Affiliations:** 1 Department of Surgery, Chi Mei Medical Center, Tainan, Taiwan; 2 Department of Food Science and Technology, Chia Nan University of Pharmacy and Science, Tainan, Taiwan; 3 Department of Pathology, Shuang Ho Hospital, Taipei Medical University, New Taipei, Taiwan; 4 Department of Pathology, School of Medicine, College of Medicine, Taipei Medical University, Taipei, Taiwan; 5 Department of Medical Research, Chi Mei Medical Center, Tainan, Taiwan; 6 Department of Pathology, Wang Fang Hospital, Taipei Medical University, Taipei, Taiwan; 7 Department of Biotechnology, Chia Nan University of Pharmacy and Science, Tainan, Taiwan

**Keywords:** gastric cancer, TMPO-α, prognosis.

## Abstract

As one of the deadliest and most common malignancies in the world, gastric cancer (GC) represents a serious health threat. Despite recent advances in the field, the prognosis of patients with metastatic GC remains poor. In this study, we aimed to investigate the clinical impact of the alpha subunit of the nuclear structural protein thymopoietin (TMPO-α) in GC. The expression of TMPO-α in seven gastric cell lines was detected by immunoblotting. The expression level of TMPO-α levels in gastric tissues collected from 145 GC patients was examined by immunohistochemistry. The correlations between TMPO-α expression level and clinicopathologic parameters, as well as the association of TMPO-α expression with overall survival, were assessed. Immunohistochemistry showed that the expression of TMPO-α was significantly higher in GC tissues and cells in comparison with non-tumor tissues and cells. Furthermore, the overexpression of TMPO-α in gastric tissues (56%) was positively associated with Lauren classification (*P* = 0.0159), nodal status (*P* = 0.0265), distant metastasis (*P* < 0.0001), stage (*P* = 0.0367), and degree of differentiation (*P* = 0.0009). Patients with high TMPO-α levels had a significantly poorer overall survival than those with low levels (*P* = 0.001). Multivariate Cox regression analysis also indicated that TMPO-α was an independent prognostic marker for GC (*P* = 0.045). In addition, studies conducted in GC cells indicated that knockdown of TMPO-α suppressed cell proliferation and invasion. These findings indicate that TMPO-α overexpression can predict clinicopathologic features and the outcome of patients with GC.

## Introduction

Gastric cancer is the third most common cause of cancer-related deaths and accounts for more than 2000 deaths per year in Taiwan (http://www.mohw.gov.tw/CHT/DOS/Statistic.aspx?f_list_no=312&fod_list_no=6201) 1. The outcomes of patients with GC remain unsatisfactory because of poor understanding of the pathogenesis and the lack of effective prognostic biomarkers 2,3. Therefore, identifying novel biomarkers for GC is of significant clinical benefit. In recent decades, improvements in molecular biology techniques have allowed for the elucidation of several GC-related molecules, including oncogenes, tumor suppressor genes, and their expressed proteins, which have been investigated for their mechanistic involvement in GC tumorigenesis, as well as for their potential as prognostic biomarkers 4-10.

Basic molecular and cellular mechanism alterations seem to be implicated in the acquisition and progress of GC 11, dysregulation of the cell cycle is one of the most important alterations in carcinogenesis. Many proteins were found to be involved in the cell-cycle regulation 12. One such cell cycle associated protein is thymopoietin (TMPO), which is also known as lamina-associated polypeptide 2 (LAP2). In mammals, TMPO family is comprised of six different isoforms (α, β, γ, δ, ε, and ζ), which are generated by alternative splicing of the same transcript and which share a common N-terminal domain. All isoforms, except TMPO-α and TMPO-ζ, are inner nuclear membrane proteins. TMPO can interact with lamins and BAF to regulate the organization of the nuclear structure and the dynamics of the cell cycle 13. Unlike the membrane bound TMPO isoforms that interact with lamin B 14, TMPO-α specifically binds to lamin A via its unique C-terminus 15 in the nucleoplasm 16. The role of TMPO in cancer biology has been reported 17. For example, the C-terminus of TMPO-α mediates interaction with pRb, a cell cycle regulator and tumor suppressor. Further observations that TMPO-α may be involved in the regulation of pRb localization and tumor suppressor activity led to the hypothesis that TMPO-α may play a role in tumorigenesis 16,18,19.

Several studies have revealed the overexpression of TMPO in different cancer types, such as gastric, pancreatic, liver, and bile duct cancer 20. At present, the α and β isoforms of TMPO are the best characterized. Parise et al. investigated the expression of TMPO-α mRNA using tissue microarray and showed that TMPO-α mRNA was up-regulated in lung, breast, colon, and gastric cancers 21. In addition, Somech and his colleagues showed that TMPO-β was up-regulated in the rapidly proliferating cells of various hematological malignancies 22. TMPO-β is also up-regulated in pancreatic cancer tissues and cells, and its knockdown not only inhibits cell proliferation but also suppresses migration, invasion, and metastasis 20.

Although the expression of TMPO in cancer has been reported, understanding of the prognostic impact of TMPO on patients with cancer has been very limited. The study performed by Gentles et al. indicated that overexpression of TMPO-α could predict the poor survival of lymphoma patients 23. Another paper reported that TMPO-α expression in myeloma was significantly associated with poor overall survival 24. To our knowledge, the value of TMPO-α as a prognostic marker for GC has not been determined.

To assess the clinical impact of TMPO-α in human GC, a patient cohort was enrolled to examine the expression of TMPO-α in gastric tissues, investigate the associations between TMPO-α expression and clinicopathologic parameters, and evaluate the feasibility of TMPO-α as a prognostic biomarker for GC patients.

## Materials and Methods

### Patients and specimens

The patient cohort comprised of 145 consecutive GC cases from 1998 through 2011 with documented pathologic and clinical factors as well as clinical outcomes. Non-tumor tissues were obtained from grossly normal gastric mucosa adjacent to the tumor in the resected gastric specimen. Clinicopathologic parameters of GCs were determined according to the American Joint Committee on Cancer (AJCC) classification. The follow-up duration for disease-free survival was defined as the period between the operation date and the day of relapse, according to the patient's chart. For each patient, we analyzed a pair of tumor and non-tumor gastric tissues to determine the TMPO-α expression. The tissue acquisition protocol for the immunohistochemical and immunoblotting study was approved by the Institutional Review Board at Wan Fang Hospital, Taipei, Taiwan (Approval No. 99049). Written informed consent was obtained from each participant before tissue acquisition.

### Immunohistochemical analysis

Paraffin-embedded tissue blocks were sliced into 5-μm sections and transferred onto microscope slides (Muto Pure Chemicals Co. Ltd., Tokyo, Japan). Normal liver was used as a positive control for the expression of TMPO-α. Deparaffinized sections were incubated in citrate buffer (pH 6.0) for 40 min at 95˚C for antigen retrieval. Primary antibody (rabbit polyclonal anti- TMPO-α antibody, Aviva, San Diego, CA) at a dilution of 1:300 was applied to the sections for 45 min at room temperature. Detection of the immunoreactive staining was conducted using the avidin-biotin-peroxidase complex method according to the manufacturer's instructions (Dako REAL EnVision Detection System, Glostrup, Denmark). After the sections were incubated with diaminobenzidine for 5 min, they were counterstained with hematoxylin and mounted in Dako Faramount Aqueous Mounting Medium for microscopic analysis. Immunoreactivity was assessed semi-quantitatively and scored as follows: 0, no staining; 1, weak and focal staining in < 25% of the tissue; 2, moderate staining in 25%-50% of the tissue; and 3, strong staining in > 50% of the tissue. Sections with a score of 0 or 1 exhibited low expression of TMPO-α, and those with a score of 2 or 3 were defined as exhibiting high expression or overexpression of TMPO-α. Clinical data collection and immunohistochemical analysis were performed independently of each other in an investigator-blinded manner.

### Cell culture

A human normal gastric cell line (Hs738.St/Int) was obtained from the American Type Culture Collection (ATCC, Manassas, VA, Cat. No. CRL-7869). Four GC cell lines (AGS, NCI-N87, TMC-1, and TSGH 9201) were obtained from the Bioresource Collection and Research Center (BCRC, Hsinchu, Taiwan, Cat. No. BCRC 60102, 60217, 60379, and 60146, respectively). The GC cell lines SK-GT-2 and HGC-27 were obtained from the European Collection of Cell Cultures (ECACC, Salisbury, UK). Cells were cultured in DMEM (Hs738.St/Int), F-12K (AGS), and RPMI-1640 (NCI-N87, TMC-1, TSGH 9201, SK-GT-2, and HGC-27) supplemented with 10% fetal bovine serum (FBS), 100 units/mL penicillin G, 100 μg/mL streptomycin sulfate, and 250 ng/mL amphotericin B.

### Total protein preparation

Total protein was extracted with the RIPA Protein Extraction Reagent (Pierce Biotechnology, Rockford, IL) according to the manufacturer's instructions. The samples were stored at -80°C until further analysis. The protein concentration was determined using a BCA Protein Assay Kit (Pierce Biotechnology) with bovine serum albumin as a standard.

### Immunoblotting

Denatured protein samples were subjected to 10% SDS-PAGE. The proteins were transferred to nitrocellulose membranes, and blocked blots were incubated with an anti- TMPO-α rabbit polyclonal antibody (Abcam, Cambridge, MA, 1:1000 dilution) overnight at room temperature. β-Actin was used as an internal control for equal protein loading. The blots were further incubated with secondary antibodies conjugated with peroxidase (Sigma, St. Louis, MO) for 1 h at room temperature. They were then incubated with Western Lighting ECL Ultra Chemiluminescence Substrate (PerkinElmer, Waltham, MA), and exposed to Fuji medical X-ray film (Fuji Photo Film Co., Tokyo, Japan). Image processing was performed using the Fuji Image Gauge software.

### siRNA treatment

For siRNA treatment, SK-GT-2 cells were transfected with siRNAs (two TMPO-α-siRNAs and one control; all purchased from Sigma), and the knockdown efficiency was examined by immunoblotting.

### Colony formation assay

Five hundred cells were seeded into six-well plates and cultured for 12 days. Individual colonies (> 50 cells/colony) were fixed, stained in a solution of 1% crystal violet in methanol, and counted. The plates were scanned with a Scanjet 2200c scanner (HP, Palo Alto, CA). After scanning, methanol was added, and the plates were shaken at room temperature to solubilize the crystal violet. The optical density (OD_540_) was measured to quantify the number of colonies formed. The assay was performed three times, and the results are presented as the mean ± the standard deviation (SD).

### In vitro invasion assay

Cell invasion was examined using a Cell Invasion Assay Kit (Merck Millipore, Darmstadt, Germany) following the manufacturer's instructions. Complete media was first added to 24-well plates. The cells (2 × 10^5^) in serum-free media were added to ECMatrix-layered cell culture inserts (containing polycarbonate membranes with an 8 μm pore size) and cultured for 24 h. Before staining, the cells on the upper surface were removed. Inserts were then stained to mark invasive cells on the lower surface of the membranes and then photographed (100× magnification, with Leica DMIRB microscope).

### Statistical analysis

Paired t test was used to assess the difference in TMPO-α expression between tumor and non-tumor tissues for each patient. We examined several clinicopathologic parameters: age, gender, depth of invasion, nodal status, distant metastasis, stage, degree of differentiation, and vascular permeation. The correlation between TMPO-α expression and each clinicopathologic parameter was examined using the χ^2^ test. The time-to-event endpoints for all clinicopathologic parameters were plotted using the Kaplan-Meier method, and the degree of significance was calculated using the univariate log-rank test. *P* < 0.05 was considered statistically significant. Parameters that emerged as significant (*P* < 0.05) in the univariate analysis were entered as variables in the multivariate Cox regression model, and the hazard ratio (HR) and the independence of prognostic impact were determined in a stepwise backward fashion. All data were analyzed using SPSS software version 24.0 (IBM, New York, NY).

## Results

### Demographics

This study enrolled 145 patients with GC; 94 were male, and 51 were female (Table 1). The patients' ages ranged from 34 to 96 years at first diagnosis (mean 69.3 years). According to the AJCC classification, 26 patients were at stage I, 37 were at stage II, 63 were at stage III, and 19 were at stage IV. The follow-up period for all patients ranged from 0 to 3498 days (mean 916 days). Ninety-four patients died during follow-up.

### TMPO-α expression was increased and associated with several clinicopathologic parameters in GC

**Figure 1:**
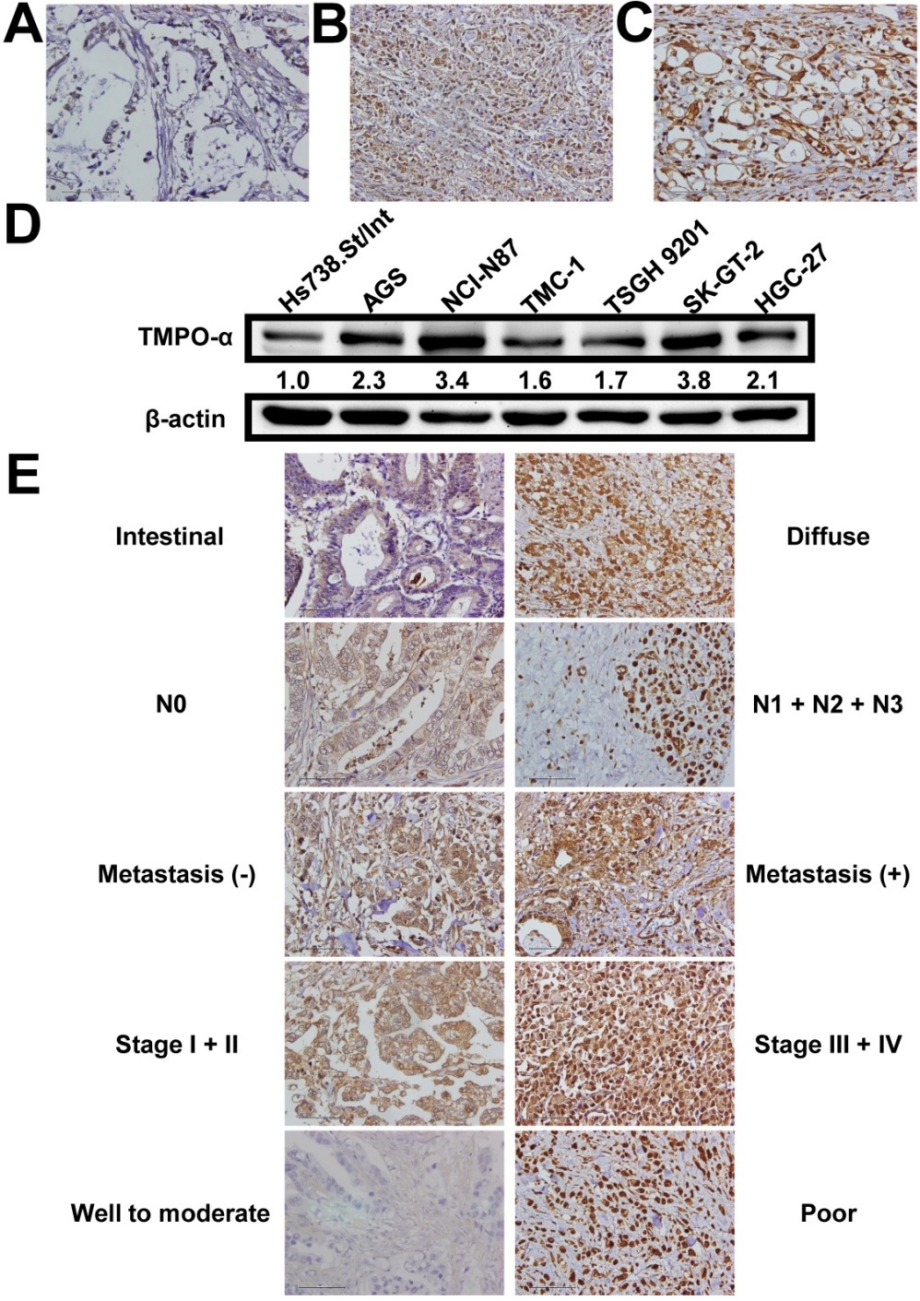
** Expression of TMPO-α in gastric tissues and cells.** TMPO-α protein expression was significantly increased in GC tissues (**A-C**) and cells (**D**). Panel **A** shows a sample of non-tumor tissue without TMPO-α expression; Panel **B** shows a tumor specimen with low TMPO-α expression; Panel **C** shows a tumor specimen with high TMPO-α expression. (**E**) The representative TMPO-α staining for different parameters.

We used immunohistochemistry to examine the TMPO-α level in tissues obtained from our study patients (Figure 1A-C). TMPO-α expression was significantly higher in tumor tissues than in non-tumor tissues (*P* < 0.001). Overexpression of TMPO-α (scores of 2 or 3) was observed in 81 (55.9%) patients in the cohort. Immunoblotting analysis showed that the expression of TMPO-α was substantially higher in GC cells than in normal cells (Figure 1D). As shown in Table 2, overexpression of TMPO-α was associated with Lauren classification, nodal status, distant metastasis, stage, and degree of differentiation (*P* = 0.0159, 0.0265, <0.0001, 0.0376, and 0.0009, respectively). Representative images of TMPO-α expression for different parameters are shown in Figure 1E. In contrast, other clinicopathologic parameters were not meaningfully correlated with the TMPO-α protein level (Table 2).

### TMPO-α overexpression is a poor prognosticator for GC

**Figure 2:**
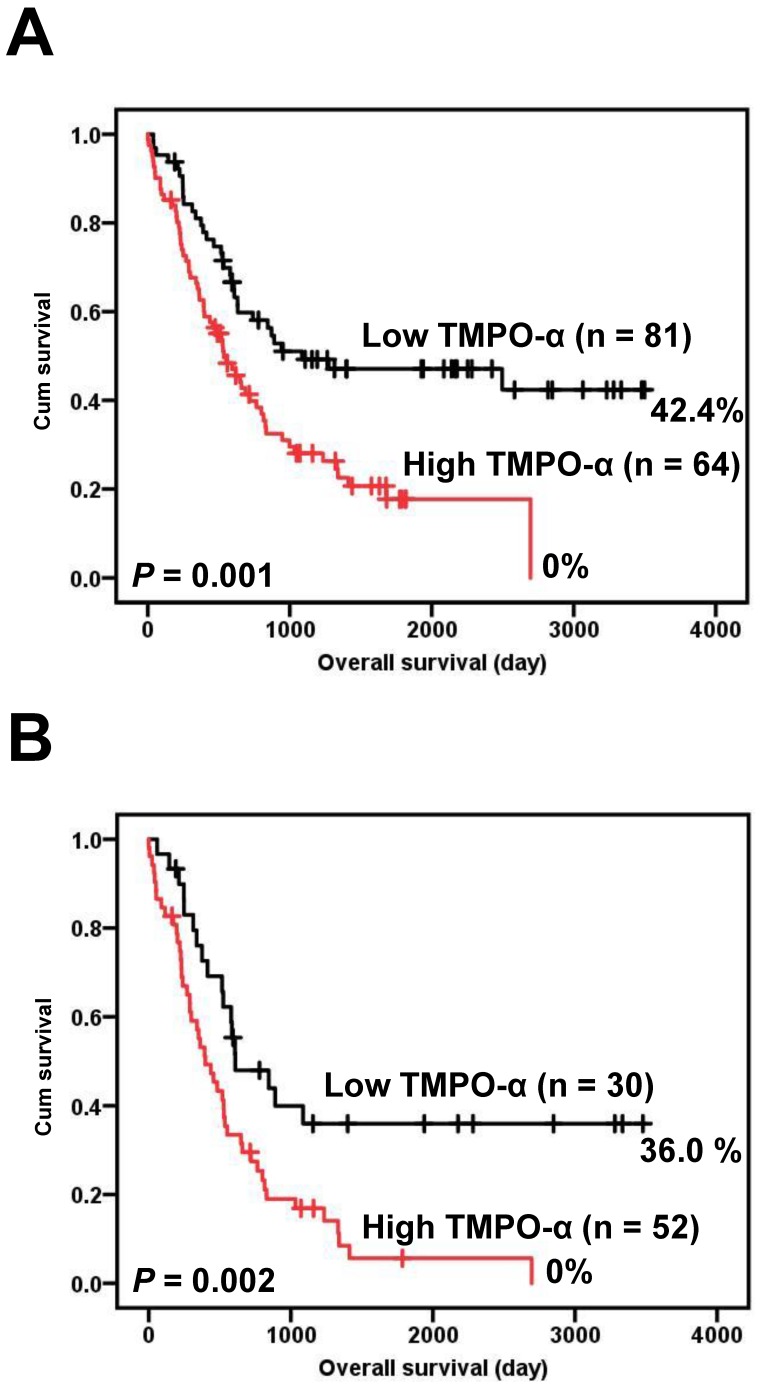
** Survival analysis of GC patients stratified by TMPO-α immunoreactivity. Panel A** shows the overall survival. Patients with high TMPO-α expression had a 5-year overall survival rate of 17.7% compared with 47.1% for patients with low TMPO-α expression. **Panel B** shows the overall survival in high-stage GC (stages III and IV). Patients with high TMPO-α expression had a 5-year disease-free rate of 5.6% compared with 36.0% for patients with low TMPO-α expression. All statistical tests were two-tailed. Significance level: *P* < 0.05.

Correlations of the clinical outcomes with TMPO-α expression level are presented in Figure 2. Overexpression of TMPO-α was significantly associated with inferior overall survival (Figure 2A, *P* = 0.001). The 5-year overall survival rate in patients with high TMPO-α expression was 17.7%, whereas the 5-year overall survival rate in patients with low TMPO-α expression was 47.1%. Tumor stage is a critical prognostic marker of GC. We stratified our GC patient cohort into low-stage (stage I and II) and high-stage GC (stage III and IV) and then independently used these classifications to determine the effect of TMPO-α overexpression on patient survival. The data showed that in high-stage GC, a shorter overall survival was significantly associated with overexpression of TMPO-α (Figure 2B, *P* = 0.002).

The results of the univariate analysis of the prognostic markers of GC are shown in Table 3. Overall survival was significantly correlated with each of the following: overexpression of TMPO-α (*P* = 0.001), age (*P* = 0.012), depth of invasion (*P* = 0.003), nodal status (*P* < 0.001), distant metastasis (*P* < 0.001), stage (*P* < 0.001), degree of differentiation (*P* = 0.022), and vascular invasion (*P* = 0.002). The association between overexpression of TMPO-α and survival was significant, even after controlling for other well-known prognostic markers in the multivariate analysis (Table 4). In the multivariate analysis, overexpression of TMPO-α (HR = 1.643, 95% confidence interval (CI) = 1.012 to 2.669, *P* = 0.045), age (HR = 3.053, 95% CI = 1.792 to 5.201, *P* < 0.001), nodal status (HR = 2.384, 95% CI = 1.183 to 4.805, *P* = 0.015), and distant metastasis (HR = 2.393, 95% CI = 1.268 to 4.516, *P* = 0.007) were prognostically independent.

### TMPO-α knockdown impaired cell proliferation and invasion in GC cells

**Figure 3:**
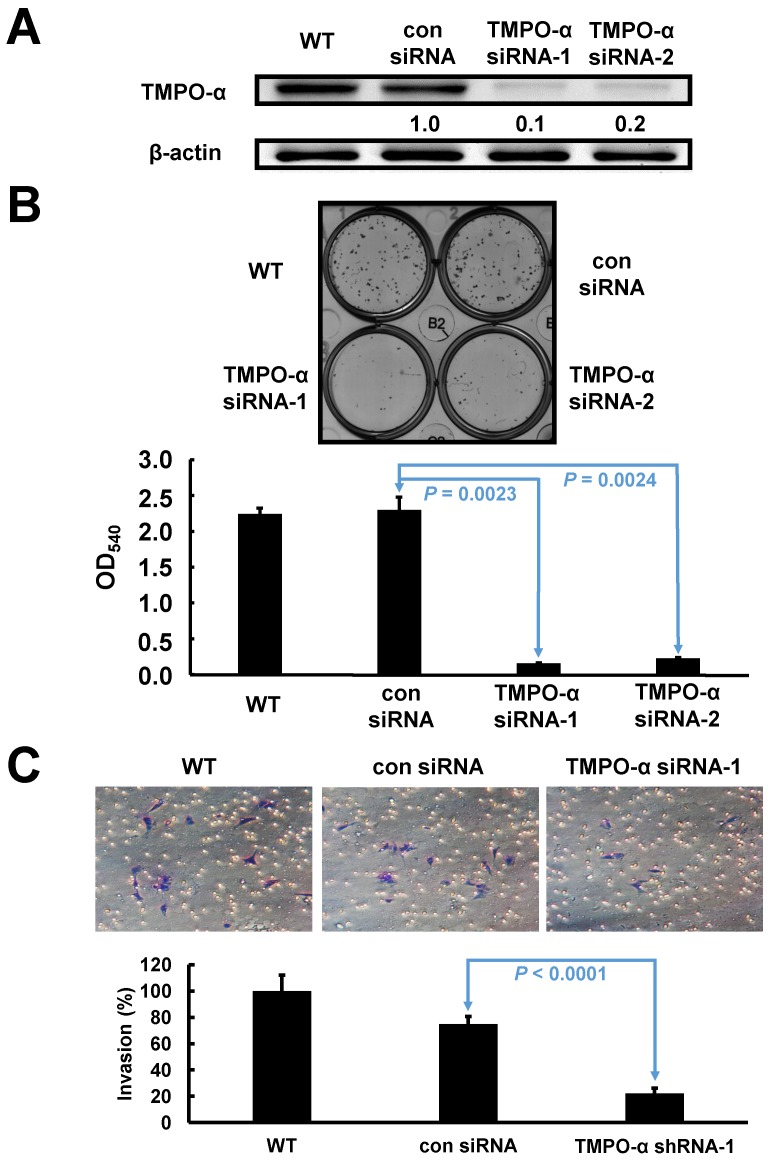
** Verification of TMPO-α knockdown in SK-GT-2 cells, and the effect of TMPO-α knockdown on cell proliferation and invasion.** The immunoblotting results (**A**) indicate that TMPO-α was efficiently knocked down by siRNA treatment. (**B**) Inhibition of TMPO-α expression suppressed cell proliferation. The histogram represents OD_540_ (presented as the mean ± SD). Significance level: *P* < 0.05. (**C**) Silencing TMPO-α expression repressed cell invasion. The histogram represents the number of invaded cells (presented as the mean ± SD). Significance level: *P* < 0.05.

Based on the endogenous high expression levels of TMPO-α, SK-GT-2 GC cells were selected to investigate the role of TMPO-α in modulating cell proliferation (Figure 1D). SK-GT-2 cells were transfected with TMPO-α-siRNA to generate TMPO-α-knockdown SK-GT-2 cells (Figure 3A). As shown in Figure 3B, the ability of SK-GT-2 cells to form colonies was compromised by TMPO-α knockdown compared with the corresponding control cells. These results suggested that TMPO-α knockdown suppressed the ability of GC cells to proliferate in vitro.

Furthermore, according to the results of the clinicopathologic correlation study-which showed that TMPO-α overexpression was closely associated with the depth of invasion and vascular invasion, the effect of TMPO-α knockdown on the invasiveness of SK-GT-2 GC cells was examined. In the cell invasion assay, TMPO-α knockdown significantly suppressed SK-GT-2 cell invasion compared with control cells (Figure 3C).

## Discussion

Exploration of novel genetic or proteomic changes that occur during GC progression is important to identify valuable prognostic biomarkers or therapeutic targets. Here, we examined the level of TMPO-α in GC, investigated the correlation between TMPO-α expression and clinicopathologic parameters, and evaluated the prognostic significance of TMPO-α expression in GC. Furthermore, RNA interference was used to study the role of TMPO-α in GC carcinogenesis.

It is well known that abnormal cell proliferation, initiated by dysregulation of the cell cycle, is a fundamental driving force of cancer 25. Many genes have been found to be involved in the regulation of the cell cycle, including TMPO-α 12. TMPO-α mRNA has been shown to be up-regulated in cancer cell lines and several types of cancer such as larynx, lung, prostate, and colon 21. By using in situ hybridization, the only published study conducted in GC demonstrated that TMPO-α mRNA was up-regulated in human GC tissues 21. Thus far, the protein level of TMPO-α in all types of cancer, including GC, is still unclear. Herein, we examined the expression of TMPO-α in GC by immunohistochemistry. The data indicated that TMPO-α expression was up-regulated in GC tissues in comparison to adjacent normal gastric tissues. To further confirm the immunohistochemical results, immunoblotting was performed to examine the expression of TMPO-α in normal and GC cells. The results also showed that TMPO-α expression was higher in GC cells than in normal gastric cells. Overall, our data were consistent with those of previous studies and demonstrated the expression of TMPO-α protein in GC for the first time. The mechanism by which TMPO is overexpressed in cancer is unclear. Parise et al. reported that the expression of TMPO is under the direct control of E2F transcription factors. Chromatin immunoprecipitation assays showed that the TMPO promoter is bound by endogenous E2F in vivo. The TMPO promoter is transactivated by ectopically expressed E2F and mutation of E2F binding sites abolished this effect 21. Whether the overexpression of TMPO-α in GC is controlled by this mechanism needs to be investigated.

Statistical analysis showed that TMPO-α was overexpressed in diffuse type and poorly differentiated GC. In vitro study also indicated that inhibition of TMPO-α in SK-GT-2 human GC cells hindered cell proliferation. It is well known that, as cells differentiate, their rate of proliferation decreases 26. The relationship between TMPO expression and cell proliferation has been investigated in several cancer types. It has been demonstrated that knockdown of TMPO can significantly inhibit glioblastoma cell proliferation and arrest cell cycle progression at the G2/M phase 27. A study by Parise et al. using human tumor tissue microarray indicated that TMPO-α expression was significantly correlated with tumor proliferation rate based on the expression of Ki67 21. Similar results obtained from hematological malignancies also revealed that the level of TMPO-β was up-regulated in rapidly replicating cells 22. In contrast, it was shown that knockdown of TMPO-β had no effect on the proliferation of gastric and liver cancer cells. The relationship between TMPO-α overexpression and cell proliferation remains controversial and warrants further investigation.

We showed, herein, that TMPO-α overexpression was positively correlated with distant metastasis. The data from the in vitro cell invasion assay further supported this correlation. The correlation between TMPO expression and metastasis has not been well established. The sole study reported by Kim et al. showed that knockdown or overexpression of TMPO-β decreased or increased the motility of gastric and pancreatic cancer cells 20. Additionally, in a liver metastasis xenograft model, TMPO-β increased metastasis of gastric cancer cells. To the best of our knowledge, our study represents the first demonstration of the positive correlation between TMPO-α expression and invasion. However, an explanation of this relationship between TMPO-α overexpression and vascular invasion remains unclear and warrants further investigation.

Assessment of clinical outcomes of GC patients is currently reliant upon AJCC stage 28. However, the prognosis varies even among patients with the same disease stage. Therefore, useful prognostic markers are urgently needed to refine the risk stratification for the prognosis of patients with GC. In the present study, we found that TMPO-α overexpression in GC was significantly associated with poor overall survival. The results were consistent with two previous studies conducted in lymphoma and myeloma 23,24. Our study is the first to report that overexpression of TMPO-α is a prognostic marker for GC. Overexpression of TMPO-α appears to be a useful marker to predict clinical outcomes of GC patient who have undergone surgical resection. Therefore, clinical management of GC patients with overexpression of TMPO-α should include frequent follow-up visits.

In summary, this study provides evidence for the clinical significance of TMPO-α overexpression in GC patients. Our findings indicate that targeting TMPO-α may provide a new therapeutic strategy for the treatment of GC.

## Figures and Tables

**Table 1:** Demographic data and survival in different stages of GC according to the AJCC classification

	Stage I (n = 26)	Stage II (n = 37)	Stage III (n = 63)	Stage IV (n = 19)	Total (n = 145)
Gender					
Male	16	24	43	11	94
Female	10	13	20	8	51
Age (years)*	68.5 (11.7)	75.7 (10.2)	69.3 (12.7)	57.4 (15.3)	69.3 (13.3)
Follow-up period (days) *	1392.0 (996.4)	981.0 (726.6)	869.7 (841.7)	332.5 (274.0)	921.4 (840.6)
Survival					
Yes	17	17	15	2	51
No	9	20	48	17	94

*Age and follow-up period are expressed as the mean (SD)

**Table 2:** TMPO-α expression in GC and its correlation with clinicopathologic parameters

Variable	n	TMPO-α expression		P*
		Score = 0 or 1 (n = 64)	Score = 2 or 3 (n = 81)	
Age (yr)				0.1996
≥ 66	96	46	50	
< 66	49	18	31	
Gender				0.5968
Male	94	43	51	
Female	51	21	30	
Lauren classification				0.0159
Intestinal	98	50	48	
Diffuse	47	14	33	
Depth of invasion				0.6950
T1 + T2	34	16	18	
T3 + T4	111	48	63	
Nodal status				0.0265
N0	45	26	19	
N1 + N2 + N3	100	38	62	
Distant metastasis				< 0.0001
Absent	126	64	62	
Present	19	0	19	
Stage				0.0367
I + II	63	34	29	
III + IV	82	30	52	
Degree of differentiation				0.0009
Poor	63	18	45	
Well to moderate	82	46	36	
Vascular invasion				0.3481
Absent	44	22	22	
Present	101	42	59	

* All statistical tests were two-tailed. Significance level: *P* < 0.05.

**Table 3:** Univariate analysis of prognostic markers in 145 patients with GC

Variable	HR (95 % CI)*	P*
TMPO-α Low expression High expression	2.077 (1.351-3.193)	0.001
Age (yr) ≥ 66 < 66	1.803 (1.138-2.856)	0.012
Gender Male Female	0.704 (0.452-1.096)	0.120
Lauren classification Intestinal Diffuse	1.233 (0.804-1.891)	0.336
Depth of invasion T1 + T2 T3 + T4	2.391 (1.352-4.228)	0.003
Nodal status N0 N1 + N2 + N3	3.247 (1.913-15.512)	< 0.001
Distant metastasis Absent Present	3.507 (2.030-6.058)	< 0.001
Stage I + II III + IV	2.519 (1.620-3.915)	< 0.001
Degree of differentiation Poor Well to moderate	0.623 (0.415-0.935)	0.022
Vascular invasion Absent Present	2.132 (1.306-3.480)	0.002

* All statistical tests were two-tailed. Significance level: *P* < 0.05. HR = hazard ratio; CI = confidence interval.

**Table 4:** Multivariate analysis of prognostic markers in 145 patients with GC

Variable	HR (95 % CI)*	P*
TMPO-α Low expression High expression	1.643 (1.012-2.669)	0.045
Age (yr) ≥ 66 < 66	3.053 (1.792-5.201)	< 0.001
Depth of invasion T1 + T2 T3 + T4	1.082 (0.515-2.274)	0.835
Nodal status N0 N1 + N2 + N3	2.384 (1.183-4.805)	0.015
Distant metastasis Absent Present	2.393 (1.268-4.516)	0.007
Stage I + II III + IV	1.390 (0.693-2.788)	0.354
Degree of differentiation Poor Well to moderate	0.666 (0.402-1.104)	0.115
Vascular invasion Absent Present	0.873 (0.484-1.574	0.651

* All statistical tests were two-tailed. Significance level: *P* < 0.05.
